# Automatic Fungi Recognition: Deep Learning Meets Mycology

**DOI:** 10.3390/s22020633

**Published:** 2022-01-14

**Authors:** Lukáš Picek, Milan Šulc, Jiří Matas, Jacob Heilmann-Clausen, Thomas S. Jeppesen, Emil Lind

**Affiliations:** 1 Department of Cybernetics, Faculty of Applied Sciences, University of West Bohemia, 30100 Pilsen, Czech Republic; 2 Department of Cybernetics, Faculty of Electrical Engineering, Czech Technical University in Prague, 16636 Prague, Czech Republic; milansulc01@gmail.com (M.Š.); matas@fel.cvut.cz (J.M.); 3 Center for Macroecology, Evolution and Climate, Biological Institute, University of Copenhagen, 1165 Copenhagen, Denmark; jheilmann-clausen@snm.ku.dk (J.H.-C.); lind@noque.dk (E.L.); 4 Global Biodiversity Information Facility, 2100 Copenhagen, Denmark; tsjeppesen@gbif.org

**Keywords:** fungi, species, classification, recognition, machine learning, computer vision, species recognition, fine-grained, artificial intelligence

## Abstract

The article presents an AI-based fungi species recognition system for a citizen-science community. The system’s real-time identification too — FungiVision — with a mobile application front-end, led to increased public interest in fungi, quadrupling the number of citizens collecting data. FungiVision, deployed with a human-in-the-loop, reaches nearly 93% accuracy. Using the collected data, we developed a novel fine-grained classification dataset — Danish Fungi 2020 (DF20) — with several unique characteristics: species-level labels, a small number of errors, and rich observation metadata. The dataset enables the testing of the ability to improve classification using metadata, e.g., time, location, habitat and substrate, facilitates classifier calibration testing and finally allows the study of the impact of the device settings on the classification performance. The continual flow of labelled data supports improvements of the online recognition system. Finally, we present a novel method for the fungi recognition service, based on a Vision Transformer architecture. Trained on DF20 and exploiting available metadata, it achieves a recognition error that is 46.75% lower than the current system. By providing a stream of labeled data in one direction, and an accuracy increase in the other, the collaboration creates a virtuous cycle helping both communities.

## 1. Introduction

The collection and annotation of data on the appearance and occurrence of species are crucial pillars of biological research and practical nature conservation work focusing on biodiversity, climate change and species extinction [1,2]. The involvement of citizen communities is a cost effective approach to large scale data acquisition. Species observation datasets collected by the public have already been proven to improve data quality and to add significant value for understanding both basic and more applied aspects of mycology [3,4,5,6]. Citizen-science contributions provide more than 50% of all data accessible through the Global Biodiversity Information Facility [7].

In citizen-science projects focusing on biodiversity, correct species identification is a challenge. Poor data quality is often quoted as a major concern about species data provided by untrained citizens [8]. Some projects handle the issue by reducing the complexity of the species identification process, for example, by merging species into multitaxa indicator groups [9], by focusing only on a subset of easily identifiable species or by involving human expert validators in the identification process. Other projects involve citizen-science communities in the data validation process. For instance, iNaturalist [10] regards observations as research-grade labelled if three independent users have verified a suggested taxon based on an uploaded photo. Automatic image-based species identification can act both as a supplement or an alternative to these approaches.

We are interested in automating the process of fungi identification using machine learning. This has been made possible by the rapid progress of computer vision in the past decade, which was, to a great extent, facilitated by the existence of large-scale image collections. In the case of image recognition, the introduction of the ImageNet [11] database and its use in the ILSVRC (The ImageNet Large Scale Visual Recognition Challenge) challenge [12], together with PASCAL VOC [13], helped start the CNN revolution. The same holds for the problem of fine-grained visual categorization (FGVC), where datasets and challenges like PlantCLEF [14,15,16], iNaturalist [17], CUB [18], and Oxford Flowers [19] have triggered the development and evaluation of novel approaches to fine-grained domain adaptation [20], domain specific transfer learning [21], image retrieval [22,23,24], unsupervised visual representation [25,26], few-shot learning [27], transfer learning [21] and prior-shift [28].

In this paper, we describe a system for AI-based fungi species recognition to help a citizen-science community — the Atlas of Danish Fungi. The system for fungi recognition “*in the wild*” achieved the best results in a Kaggle competition sponsored by the Danish Mycological Society, which was organized in conjunction with the Fine-Grained Categorization Workshop at CVPR 2018. The real-time identification tool (FungiVision) led to an increase in public interest in nature, quadrupling the number of citizens collecting data. It supports hands-on learning, much as children learn from their parents by asking direct and naïve questions that are answered on the spot. A supervised machine learning system with a human in the loop was created by linking the system to an existing mycological platform with an existing community-based validation process.

From the computer vision perspective, the application of the system to citizen-science data collection creates a valuable continuous stream of labelled examples for a challenging fine-grained visual classification task. Based on observations submitted to the Atlas of Danish Fungi, we introduce a novel fine-grained dataset and benchmark, the Danish Fungi 2020 (DF20). The dataset is unique in its taxonomy-accurate class labels, small number of errors, highly unbalanced long-tailed class distribution, rich observation metadata, and well-defined class hierarchy. DF20 has zero overlap with ImageNet, allowing unbiased comparison of models fine-tuned from publicly available ImageNet checkpoints. The proposed evaluation protocol enables testing the ability to improve classification using metadata — for example, precise geographic location, habitat and substrate, facilitates classifier calibration testing, and finally allows us to study the impact of the device settings on the classification performance.

Finally, we present a substantial upgrade of the first version of the fungi recognition service by: (i) shifting from CNN towards Vision Transformers (ViT), we achieved state-of-the-art results in fine-grained classification; (ii) utilizing a simple procedure for including metadata in the decision process, improving the classification accuracy by more than 2.95 percentage points, reducing the error rate by 15%; (iii) increasing the amount of training data obtained with the help of the online identification tool. A new Vision Transformer architecture, which lowers the recognition error of the current system by 46.75%, is under review before deployment. By providing a stream of labeled data in one direction, and an improvement of the FungiVision in the other, the collaboration creates a virtuous cycle that helps both communities.

This paper is an extended version of our two papers published in WACV 2020 [29] and WACV 2022 [30].

## 2. Related Work

This section introduces the fine-grained image recognition problem, describes existing community-based image collections and platforms, reviews relevant publications about machine learning for fungi recognition, and evaluates existing mobile and web applications for fungi recognition *"in the wild"*.

### 2.1. Fine-Grained Image Classification

The task of image-based fungi recognition is an instance of a fine-grained visual classification (or categorization) problem. Fine-grained image classification has progressed significantly with the emergence of very deep convolutional neural networks (DCNN), after the success of Krizhevsky’s architecture [31] in the ImageNet ILSVRC-12 competition — the ImageNet dataset itself contains a number of categories from the *Fungi* kingdom. Recent FGVC approaches are based on discriminative region detection [32,33,34], visual attention [35,36], multiple neural networks or bilinear architectures [37,38] and transfer learning [39,40]. Moreover, methods based on CNNs perform well in multiple fine-grained species identification tasks, including plant species classification [41,42,43,44], dog classification [45], snake classification [46], bird classification [18,47,48] and in general species classification [17,49,50].

### 2.2. Community-Based Image Collection and Identification

The **Global Biodiversity Information Facility** (GBIF) [51] is the largest index of biodiversity data in the world. GBIF is organized as a network involving 61 participating countries and 40 organisations (mainly international) publishing more than 62,400 biodiversity datasets under open source licenses. The index contains more than 1.9 billion species’ occurrence records of which more than 88 million include images. With the recent advances in the use of machine vision in biodiversity related technology, GBIF intends to facilitate collaborations in this field, promote responsible data use and good citation practices. GBIF has the potential to play an active role in preparing training datasets and making them accessible under open source licenses [52].

**iNaturalist** [53] is a pioneering community-based platform allowing citizen scientist and experts to upload and categorize observations of the world’s fauna, flora and fungi. iNaturalist covers more than 345,000 species through almost 85 million observations. All annotated data are directly uploaded to GBIF once verified by three independent users.

**Wild Me** is a non-profit organization that aims to combat extinction with citizen-science and artificial intelligence. Their projects using computer vision [54] to boost detection and identification include: **Flukebook**, a collaboration system to collect citizen observations of dolphins and whales and to identify individuals, **GiraffeSpotter**, a photo-identification database of giraffe encounters and many more.

The **Atlas of Danish Fungi** (Danmarks Svampeatlas) [55,56,57] is a citizen-science project that currently involves more than 3900 volunteers and contains approximately 1 million quality-checked observations of fungi. The project and its data annotation process is described in more detail in Section 3.1.

### 2.3. Machine Learning for Fungal Recognition

Machine learning and computer vision techniques are rapidly developing as tools to enhance mycological research and citizen science, but has so far mainly been used in real applications for the classification of microscopy images of fungal spores [58,59,60]. Tahir et al. [58] introduced a dataset of 40,800 labelled microscopy images of six fungal infections and proposed a method to speed up medical diagnosis, avoiding additional expensive biochemical tests. De Vooren et al. [61] published an image analysis tool for mushroom cultivars identification, analyzing morphological characters such as length, width and other shape descriptors. Zielinski et al. [60] used various CNN architectures and bag-of-words approach to classify microscopic images of ten fungi species, making the last stage of biochemical identification redundant. Thus, reducing costs and time necessary for the identification. Another classical application has aimed to understand mycelial growth patterns in order to understand fungal dynamics and interactions at the cellular level [62]. More recently, the interest in using AI as a tool to help citizen scientists and students to identify mushrooms has expanded, but so far with rather few real life applications.

#### Mobile Applications

A high number of mobile applications for fungi species identification include a computer vision classification system, mostly with positive user reviews about the AI-powered identification performance. The Picture Mushroom provides paid expert verification. None contributes data to GBIF nor to mycologists. Examples of apps with positive user reviews are the following:

- **Mushroom Identificator** with 1M+ downloads and a review score of 4.3/5, recognizing 900 fungi species — https://play.google.com/store/apps/details?id=com.pingou.champignouf (accessed on 27 October 2021);
- **Mushrooms App** with 1M+ downloads and a review score of 4.2/5, recognizing 210 fungi species — https://play.google.com/store/apps/details?id=bazinac.aplikacenahouby (accessed on 27 October 2021);
- **Mushroomizer** with 10k+ downloads and a review score of 4.3/5, recognizing 530 fungi species — https://play.google.com/store/apps/details?id=my.tensorflow.lite.examples.classification (accessed on 27 October 2021);
- **Picture Mushroom** with 1M+ downloads and a review score of 4.2/5. No record about supported species was found — https://play.google.com/store/apps/details?id=com.glority.picturemushroom (accessed on 27 October 2021).

## 3. Data

**Figure 1:**
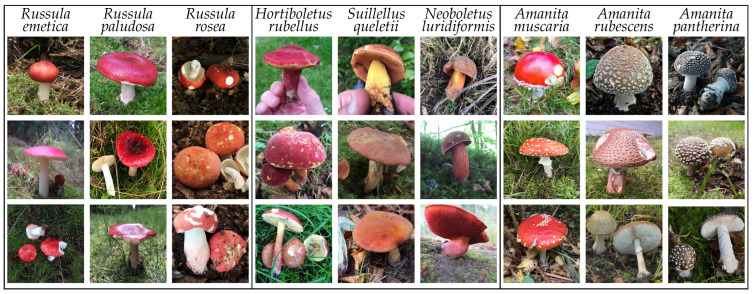
Examples of intra- and inter-class similarities and differences. Species selected from three taxonomically distinct *Fungi* families — left: *Russulaceae*, center: *Boletaceae*, right: *Amanitaceae*.

All experiments were based on datasets collected from the Atlas of Danish Fungi, which is described in Section 3.1. The details of the particular dataset are presented in Section 3.2, Section 3.3 and Section 3.4. Quantitative parameters of the used datasets are summarized in Table 1. For reference, the table includes iNaturalist 2021, the richest (in the number of species) and largest (in the number of observations) publicly available fungi dataset not based on the Atlas of Danish Fungi. The species from the *Fungi* kingdom are, by nature, visually similar, thus introducing a challenging machine learning problem. The existing high intra- and inter-class similarities and differences present in the data are visualized in Figure 1.

### 3.1. Atlas of Danish Fungi

The Atlas of Danish Fungi [55,56,57] is supported by more than 4000 volunteers who have contributed more than 1 million content-checked observations of approximately 8300 fungi species, many with expert-validated class labels. The project has resulted in a vastly improved knowledge of fungi in Denmark [57]. More than 180 species belonging to *Basidiomycota* — a group of fungi that produces their sexual spores (basidiospores) on club-shaped spore-producing structures (basidia) supported by macroscopic fruit bodies including toadstools, puffbals, polypores and other types — have been added to the list of known Danish species in the first atlas period (2009–2013) alone [57]. In addition, several species that were considered extinct were re-discovered [63]. In the second project period (2015–2022) several improved search and assistance functions have been developed that present features relating to the individual species and their identification [63], making it much easier to include an understanding of endangered species in nature management and decision-making.

#### Annotation Process

Since 2017, the Atlas of Danish Fungi has had interactive labelling procedure for all submitted observations. When a user submits a fungal sighting (record) at species level, a “reliability score” (1–100) is calculated based on following factors:

- Species rarity, that is, its relative frequency in the Atlas;
- The geographical distribution of the species;
- Phenology of the species, its seasonality;
- User’s historical species-level proposal precision;
- As above, within the proposal’s higher taxon rank.

Subsequently, other users may agree with the proposed species’ identity, increasing the identification score following the same principles, or proposing alternative identification for non-committal suggestions. Once the submission reaches a score of 80, the label (identification) is considered approved by community validation. Simultaneously, a small group of taxonomic experts (expert validators) monitor most of the observation on their own. Expert validators have the power to approve or reject species identifications regardless of the score in the interactive validation. Community-validated and expert-validated Svampeatlas records are published in the GBIF, weekly, since 2016. As of the end of October 2021, the data in GBIF included 955,392 occurrences with 504,165 images [64]. Since 2019, the Atlas of Danish Fungi observation identification has been further streamlined thanks to an image recognition system [29] — FungiVision.

### 3.2. The FGVCx Fungi Dataset

The FGVCx Fungi Classification Challenge provided an image dataset covering 1394 fungal species and is split into a training set with 85,578 images, a validation set with 4182 images, and a competition test set of 9758 images without publicly available labels. There is a substantial change of categorical priors p(k) between the training set and the validation set: The distribution of images per class is highly unbalanced in the training set, while the validation set distribution is uniform.

### 3.3. The Danish Fungi 2020 Dataset

The **Danish Fungi 2020** (DF20) dataset contains image observations from the Atlas of Danish Fungi belonging to species with more than 30 images. The data are observations collected before the end of 2020. Note that this includes more than 15 months of data collection using our automatic fungal identification service described later in Section 5. The dataset consists of 295,938 images represent 1,604 species mainly from the *Fungi* kingdom with a visually similar species. Unlike most computer vision datasets, DF20 include rich metadata acquired by citizen-scientists in the field while recording the observations that opens promising research direction in combining visual data with metadata like timestamp, location at multiple scales, substrate, habitat, taxonomy labels and camera device settings.

The DF20 datasets were randomly split — with respect to the class distribution — into the provided training and (public) test sets, where the training set contains ⌈90%⌉ of images of each species.

### 3.4. Test Observations from 2021

To independently compare models trained on the FGVCx Fungi dataset and the DF20, we used all validated observations submitted to the Atlas of Danish Fungi between 1 January 2021 and 31 October 2021. Only submissions that used the FungiVision system [29] were used; we choose only the first image for each observation. With this approach, we ended up with a test set of 14,391 images belonging to 999 species. In the following text, we will denote this dataset as *DanishFungi 2021* or *DF21* in short.

## 4. Methods

In this section, we describe the design of the first generation of our fungi recognition system, FungiVision, which achieved the best results in the FGVCx Fungi’18 recognition challenge. It was also applied to the Atlas of Danish Fungi, further described in Section 5. Furthermore, the evaluation of state-of-the-art classifiers on fungi data is presented. Finally, we describe a simple method for metadata integration that significantly improves the recognition capability.

### 4.1. The Baseline–FungiVision Post FGVCx Fungi’18 Competition

Following the advances in deep learning for fine-grained image classification, we decided to approach fungi recognition with Convolutional Neural Networks. For the FGVCx Fungi Classification challenge, we trained an ensemble of six models (listed in Table 2) based on Inception-v4 and Inception-ResNet-v2 architectures [65], and inspired by our winning submission in the ExpertLifeCLEF plant identification challenge 2018 [41].

All models were fine-tuned from the publicly available ImageNet-1k checkpoints using the Tensorflow Slim [66] deep learning framework. Hyper-parameters used during training were set as follows: *Optimizer*: RMSprop, *Batch Size*: 32, *Learning Rate*: 0.01, *Learning Rate Decay*: staircase with exponential decay factor 0.94, *Weight Decay*: 0.00004.

During training we used Polyak averaging [67] with *Moving Average Decay* of 0.999 to keep shadow variables with exponential moving averages of the trained variables. The six fine-tuned networks are publicly available at https://github.com/sulc/fungi-recognition.

#### 4.1.1. Adjusting Predictions by Class Priors

Unlike in the benchmark datasets — with known class priors in both training and test data — the applied machine-learning systems should be robust to different species distributions, for example, depending on seasonality, location and altitude. We utilize the following method for the adjustment of the class priors.

Let us assume that the classifier trained by cross-entropy minimization learned to estimate the posterior probabilities, that is, $f_{\mathrm{CNN}}\left(k|x\right)\approx p\left(k|x\right)$. If the class prior probabilities p(k) change, the posterior probabilities will in general change as well. The topic of adjusting CNN predictions to new priors is discussed in [28,68,69]: in the case when the new class priors $p_{e}\left(k\right)$ are known, the new posterior $p_{e}\left(k|x\right)$ can be computed as:

(1) $$\begin{array}{cc} p_{e}\left(k|x_{i}\right) & =p\left(k|x_{i}\right)\frac{p_{e}\left(k\right)p\left(x_{i}\right)}{p\left(k\right)p_{e}\left(x_{i}\right)}\\ \; & =\frac{p\left(k|x_{i}\right)\frac{p_{e}\left(k\right)}{p(k)}}{\sum_{j=1}^{K}p\left(j|x_{i}\right)\frac{p_{e}\left(j\right)}{p(j)}}\propto p\left(k|x_{i}\right)\frac{p_{e}\left(k\right)}{p(k)}, \end{array}$$

where we used $\sum_{k=1}^{K}p_{e}\left(k|x_{i}\right)=1$ to get rid of the unknown probabilities $p\left(x_{i}\right),p_{e}\left(x_{i}\right)$.

While others [28,68,69] focus on estimating new unknown priors $p_{e}\left(k\right)$, we assume that the uniform distribution $p_{e}\left(k\right)=\frac{1}{K}$ is given, as it is the case of the FGVCx Fungi’18 validation set (see Section 3.2). Then:

(2) $$p_{e}\left(k|x_{i}\right)\propto \frac{p(k|x_{i})}{p(k)}.$$

#### 4.1.2. Test-Time Image Augmentation

Let us first validate the CNN architectures listed in Table 2 on the FGVCx Fungi’18 validation set. We compare six trained models — based on two architectures Inception-v4 and Inception-ResNet-v2 — before applying additional tricks, with one feed forward pass (central crop, 80%) per image. We will continue the validation experiments with CNN 1, that is, Inception-v4 fine-tuned from an ImageNet-1k checkpoint, which achieved the best validation accuracy.

The test-time pre-processing of the image input makes a noticeable difference in accuracy. Thus, we evaluate the performance dependence for various central crop areas of the original image, various input sizes and pre-trained checkpoints. Dependence on the central crop area in terms of Top1 and Top5 accuracy is listed in in Table 3. We include two DNN architectures — Inception-v4 and ViT-Large/16 — and we provide validation on two datasets, Danish Fungi 2021 and FGVCx Fungi’18.

For a final submission, we considered the following 14 image augmentations at test time: the original image; additional six crops of the original image with 80% (central crop) and 60% (central crop + 4 corner crops) of the original image width/height; and the mirrored versions of the seven foregoing augmentations. All augmentations are then resized to square inputs using bilinear interpolation. Predictions from all crops were combined by averaging (sum) or by choosing the most common top1 prediction (mode).

The benefit of adjusting the predictions with the new categorical prior is shown in Table 4. We show that after the training, the accuracy increases by 3.8%, from 48.8% to 52.6%.

**Table 2:** Performance of different models trained on the FGVCx Fungi’18 dataset — Top1 and Top5 accuracy on the FGVCx Fungi’18 validation set; 80% central crop.

ID	Architecture	Input Size	Pretrained CKPT	Top1 [%]	Top5 [%]
1	Inception-v4	299 × 299	ImageNet-1k	**48.8**	**77.0**
2	Inception-v4	299 × 299	LifeCLEF 2018	48.5	75.8
3	Inception-v4	598 × 598	ImageNet-1k	48.6	76.6
4	Inception-v4	598 × 598	LifeCLEF 2018	48.6	76.6
5	Inception-ResNet-v2	299 × 299	ImageNet-1k	48.8	76.2
6	Inception-ResNet-v2	299 × 299	LifeCLEF 2018	47.4	75.8
[70]	Inception-v4	299 × 299	–	44.7	73.5

**Table 3:** Dependence on the central crop area. Top1 and Top5 accuracy of Inception-v4 and ViT-Large/16 on two datasets — FGVCx Fungi’18 (Validation) and Danish Fungi 2021, respectively.

	Inception-v4		ViT-Large/16	
Central Crop	Top1 [%]	Top5 [%]	Top1 [%]	Top5 [%]
100%	45.9	75.1	82.43	93.62
**80**%	**48.8**	**77.0**	**82.99**	**93.77**
60%	48.6	76.3	81.73	93.26
40%	43.1	69.3	75.03	89.24
Dataset	FGVCx Fungi’18		Danish Fungi 2021	

**Table 4:** Top1 recognition accuracy on the FGVCx Fungi validation set of a single CNN (ID 1) and ensembles (ID 1-6) with either a single central (1) or multiple crops (14). Predictions from ensembles and crops were combined by averaging (sum) or by choosing the most common top prediction (mode). Results are shown both before and after adapting to known $p_{e}\left(k\right)$.

			Baseline	Known $p_{e}\left(k\right)$
# of CNNs	Crops	Pool	Top1 [%]	Top1 [%]
1	1	–	48.8	52.6
1	14	sum	51.8	56.0
6	1	sum	54.1	58.5
6	14	sum	**54.2**	**60.3**
6	14	mode	**54.2**	59.1

### 4.2. State-of-the-Art NN Classifiers

**Figure 2:**
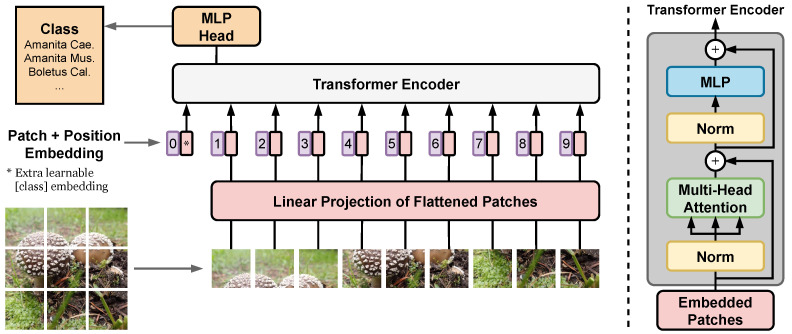
Vision Transformer architecture — main blocks. First, image is split into fixed-size patches and flatten. Second, position embeddings is added, and resulting sequence of vectors is forwarded to a standard Transformer encoder. The illustration inspired by [75].

We consider several state-of-the-art image classification architectures, which have the potential to improve the accuracy over the first generation of the FungiVision system described above. First, we choose a variety of state-of-the-art CNN architectures: SE-ResNeXt-101-32x4d [71,72], EfficientNet-B3 [73], and EfficientNetV2-L [74]. Next, we use the recently introduced Vision Transformers (ViT) [75], which showed excellent performance in object classification compared to state-of-the-art convolutional networks. Unlike CNNs, the ViT is not using convolutions, but interprets an image as a sequence of patches and processes it by a standard Transformer encoder as used in natural language processing [76] — the ViT architecture overview is described in Figure 2. Below, we describe the setup of the methods used for experiments in Section 6.3.

#### 4.2.1. Training Strategy

All architectures were initialized from publicly available ImageNet-1k pre-trained checkpoints and were further fine-tuned with the same strategy for 100 epochs with the PyTorch framework [77] within the 21.09 NGC deep learning framework Docker container. All neural networks were optimized by Stochastic Gradient Descent with momentum set to 0.9. The start Learning Rate (LR) was set to 0.01 and was further decreased with a specific adaptive learning rate schedule strategy — if the validation loss is not reduced for two epochs in a row, reduce Learning Rate by 10%. To have the same effective mini-batch size of 64 for all architectures, we accumulated gradients from smaller mini-batches accordingly, where needed.

#### 4.2.2. Augmentations

While training, we utilized several augmentations from the Albumentations library [78]. All methods, their description, and specified non-default parameters are as follow:

- *RandomResizedCrop*: Creates random resized crop with a scale of 0.8–1.0.
- *HorizontalFlip*: Flips the image horizontally with 50% probability.
- *VerticalFlip*: Flips the image Vertically with 50% probability.
- *RandomBrightnessContrast*: Changes the contrast and brightness on a given image by a random factor in a range −0.2–0.2 with 20% probability.

To match the input resolutions of the pre-trained models, all images were resized to the required network input sizes of 224 × 224 and 384 × 384. Furthermore, we re-scaled all image pixel values from 0–255 to 0–1, and we normalized it by mean (0.5) and std (0.5) values in each channel.

### 4.3. Metadata Use

We propose a simple method for the use of metadata to improve the categorization performance — similar to the spatio-temporal prior used in [79]. For a given type of metadata (*d*) and image (*i*), we adopt the following assumption for the likelihood of an image observation to get the probability of species (*s*):

(3) p(i|s)=p(i|s,d),

that is, that the visual appearance of a species (*s*) does not depend on the metadata. This does not mean that the posterior probability of a species given an image is independent of metadata *d*.

A few lines of algebraic manipulation prove that, under assumption Equation (3), the class posterior given the image *I* and metadata *D* is easily obtained:

(4) $$\begin{array}{cc} p(s|i,d) & =p\left(s|i\right)\frac{p(s|d)}{p(s)}\frac{p(i)}{p(i|d)}\propto p\left(s|i\right)\frac{p(s|d)}{p(s)}, \end{array}$$

where p(s) is the class prior in the training set. The discrete conditional probability p(s|d) is estimated as the relative frequency of species (*s*) with metadata (*d*) in the training set.

While we know this assumption is not always true in practice, since metadata, such as substrate or time, in fact do impact the image background as well as the appearance of the specimen, this is the only possible approach not requiring modelling the dependence of visual appearance and the metadata. The model trained without metadata has no information about the visual appearance changes of a species as a function of *d*. Moreover, this assumption is applicable for situations where the classifier has to be treated as a black box without the possibility of retraining the model. Even this simplistic model based on an unrealistic assumption reduces error rates, as shown later in Section 6.4.

With multiple metadata at once, for example, substrate and habitat or substrate and month, we combine the posteriors assuming statistical independence:

(5) $$p\left(s|d_{1},d_{2}\right)\propto \frac{p\left(s|d_{1}\right)p\left(s|d_{2}\right)}{p(s)}.$$

This is a simple, baseline assumption, which again may not always be valid for related metadata. Direct estimation of $p(s|d_{1},d_{2})$, for example, as relative frequencies, is another possibility. The D20 benchmark has thus the potential to be a fertile ground for evaluation of intra-metadata, as well as visual-metadata, dependencies.

The approach of Equation (4) needs a probabilistic classifier to serve as an estimator of p(s|i). In our experiments, we use the outputs of the softmax layer. Note that, for CNNs, the estimates of maxp(s|i) are typically overconfident, and the quality of the estimator can be improved by *calibration* [80,81]. The proposed benchmark allows to study new techniques for metadata integration and to domain transfer or classifier calibration.

## 5. The Application

This section describes the two main blocks of the developed image recognition pipeline, the mobile application (available for both, Android and iOS) and the classification service via Representational State Transfer (REST) API.

### 5.1. Online Fungi Classification Service

In order to provide a flexible and scalable image-based fungi identification service for the Atlas of Danish Fungi, we created a recognition server based on the open-source TensorFlow Serving [82] framework. The server currently uses one of our pretrained models, the framework allows us to deploy several models at the same time. No test-time augmentations are currently used in order to prevent server overload.

**Figure 3:**
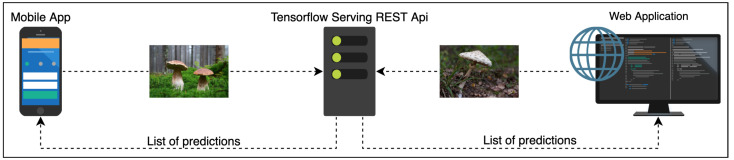
The fungi recognition serving pipeline.

The pipeline is visualized in Figure 3: The web- and mobile apps query the recognition server via REST API. The server feeds the query image into the Convolutional Network and responds with the list of predicted species probabilities. The apps then display a shortlist of the most likely species for the query. The observation might be uploaded into the database of the Atlas of Danish Fungi. The user can manually inspect the proposed species and select the best result for annotation of the fungus observation.

Observations uploaded into the Atlas of Danish Fungi database and the proposed species identifications are then verified by the community. Images with verified species labels will be used to further fine-tune the recognition system.

### 5.2. Mobile App

The foundation of the Atlas of Danish Fungi lies in the user-generated observations of Fungi and the possibility to validate their species proposals.In addition to the web-based recognition app [83], we have developed a mobile applications [84,85] with easy access to the essential functionalities of its web counterparts, including automatic fungi recognition. This section includes a detailed breakthrough of the app and how its interface affords communal contribution to the collection, identification and validation of fungi observations. It is of interest to the validity of the recorded data that it captures as much metadata to the observation as possible, which is what the application aims to simplify and automate for the user.

#### 5.2.1. Name Suggestions — Image-Based Recognition

The *Name Suggestions* feature is available regardless of whether the user is logged in or not. It is equivalent to the web-based recognition app, although this mobile version has a direct native implementation with the on-device camera.

**Figure 4:**
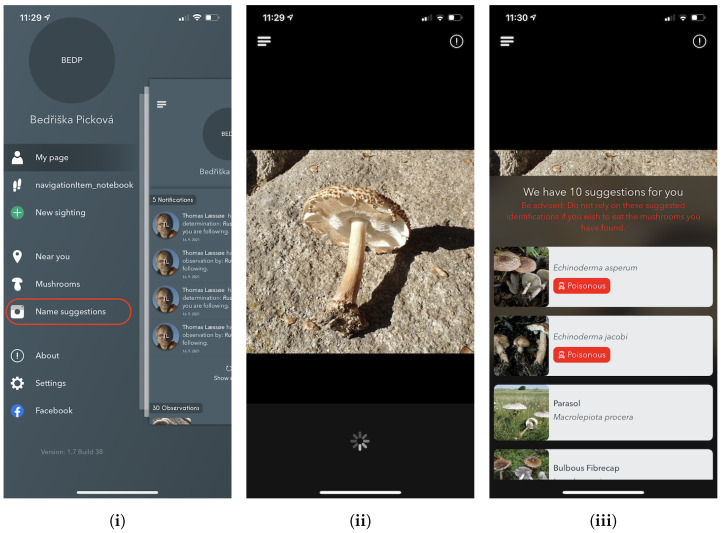
Screenshots from the iPhone app of the Atlas of Danish Fungi: (**i**) the application menu with the *Name suggestions* feature, (**ii**) image selection, and (**iii**) the fungi species suggestions with mushroom edibility info.

As shown in Figure 4, the *Name Suggestions* section offers a simple page view with their current camera viewfinder and a couple of overlays. In the upper overlay, the user can either go back using the navigation button or press the information button, which provides information about the system and how it works. For direct identification, a user can choose any photo from the image library or press the centre button to capture an image.

Upon either capturing or selecting an image, the image is then sent to the identification server (as described in Section 5.1) for processing. This requires that the user has access to the internet, although in an unpublished version of the app, a lightweight version of the model runs locally using Googles ML Kit [86].

Once the online fungi classification service processes the image, the user is presented with the ten most likely predictions. As this app is publicly available and could potentially end up in the hands of users more interested in the recognition functionality to classify edible species from non-edible species, a disclaimer is always shown advising users not to rely on the results for that use-case. Likewise, the app does not show probability scores because of fears that high probability scores could mislead users to mistakenly trust the system too much and potentially end up eating a toxic mushroom. Furthermore, if the suggested species list has all probabilities lower than 0.5, we notify the user about the prediction uncertainty that refers to unknown species or missing mushrooms on the picture.

Upon selecting one of the predictions, the app navigates to a page with details about the species, including multiple images (if available), allowing users to confirm the proposed species prediction. This page is described in Section *Species Details*.

#### 5.2.2. Species Details

The details about the previously selected species are shown on the *Species Details* page. We include: a variety of photographs, localized names in four languages (Latin, Danish, English and Czech), species descriptions, national Danish red list status (https://www.redlist.au.dk/), other observations of the same species, near observations through the map, and the possibility of submitting new fungi observations (sightings).

If the database contains more than one image, the images are shown in a carousel-like view, automatically advancing to the next image after a fixed amount of time. Moreover, we provide Latin and localized names and Danish red list status.

**Figure 5:**
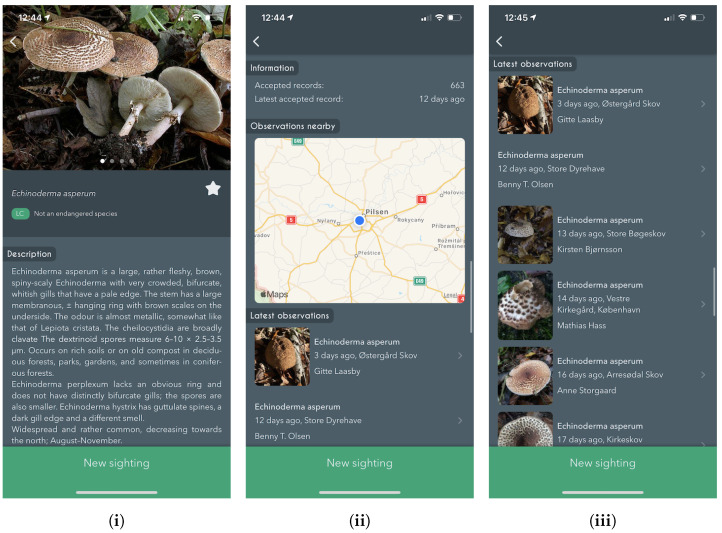
Screenshots from the iPhone app showing the *Species details* page. Including, (**i**) details about the species with multiple images, (**ii**) nearby observations of the same species on a map, and (**iii**) latest observations of the same species.

Following is a section containing 0–3 separate descriptive details about the species. As seen in Figure 5 a general textual description belongs to that specific species; other species might also contain information about the ecology and gastronomical features. Statistics regarding the number of observations recorded in the database and their last observed date are also shown. Additionally, we present a map showing all nearby observations as a heat-map, with a list of all recent observations below.

#### 5.2.3. New Sighting

The *New sighting* functionality is the main feature of the mobile application. It provides an easy and approachable method for reporting and collecting observations of Fungi to the logged-in users. We provide two ways how to submit the fungi observation. First, selecting it from the menu launches a native implementation of the mobile device’s rear-facing camera. Second, choosing to report an identification based on the *Name Suggestion* feature and transferring both the images and user based species identification.

**Figure 6:**
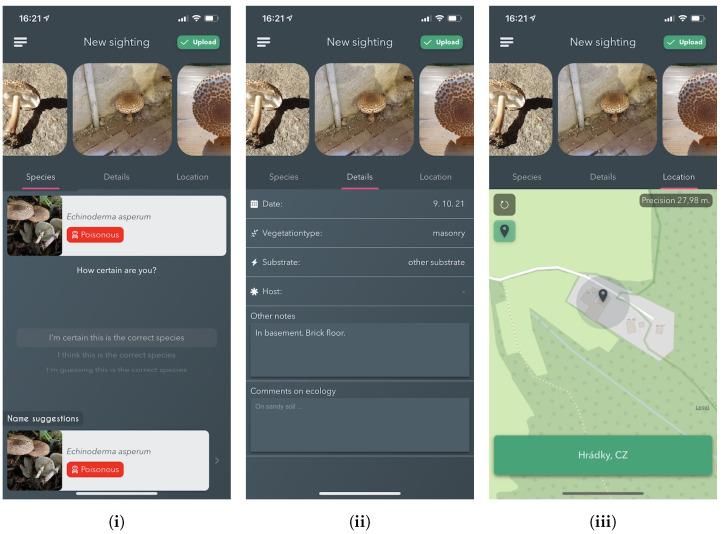
Screenshots from the iPhone app showing the process for new sighting submission. Including, (**i**) Species selection based on AI proposal, (**ii**) Observation Details selection, and (**iii**) Sighting Location Selection.

The top section of the view contains observation images, along with a "*call-to-action*" button that, when pressed, validates if the user has entered sufficient data about the sighting and then uploads it if it passes. The bottom part of the page contains a tabbed view, which stores three separate views that group the requested metadata — See Figure 6.

**Details**: Allows the entering of specific metadata about the observation. Namely, the observation date, vegetation type, substrate, hosts selections and textual information. Information about the vegetation type and the substrate is required; thus, an error pops up if a sighting is submitted without it.

**Species selection**: This sub-view allows users to search, view and select the species that is believed to be the one corresponding with what has been found. There are multiple ways in which the app aids the user in selecting. Firstly, the user can mark specific species as a favourite, thus becoming easily selectable on subsequent observations. Secondly, it automatically sends added images to the online fungi classification service for processing. The results of the processing are then shown directly in the species selection view. Every time a species is selected, either by using the results from the identification service or not, the user can select how confident they feel about the selection. If the user chooses a species from the identification suggestions, that information is embedded into the data uploaded to the server when the user is ready.

**Location**: Lastly, the location view allows the specification of the location of the observation. Upon entering the *New sighting* feature, the user’s location is automatically located by using the phone’s in-built GPS, ensuring that it is not required by the user to find the location on a map manually. Suppose an image is added with a different location included in its EXIF than the user’s current location; the user is asked if they want to use that location instead. This serves to aid the user in situations where they have captured images of sightings on either external cameras or just using the in-built camera instead of the application.

Once the user is ready to submit their sighting, they press the Upload button as explained above. Once the upload is successful, any user can validate or reject the proposed species identification for that observation.

## 6. Results

### 6.1. Machine Learning for Fungi Recognition in 2018

The FGVCx Fungi’18 competition test dataset on Kaggle was divided into two parts — public and private. Public results were calculated with approximately 70% of the test data, which were visible to all participants. The rest of the data were used for final competition evaluation to avoid bias towards the test images’ performance.

We chose our best performing system, that is, the ensemble of the six fine-tuned CNNs with 14 crops per test image and with predictions adjusted to new class priors, for the final submission to Kaggle. The accumulation of predictions was done by the mode from Top1 species per prediction as it had better preliminary scores on the public part of the FGVCx Fungi’18 test set.

Our submission to the challenge achieved the best scores in Top3 accuracy for both public and private leaderboards. The results of the top five teams are listed in Table 5.

### 6.2. Online Classification Service

The experts behind the Atlas of Danish Fungi have been highly impressed by the performance of the system; in the application, the results of the system are referred to as an AI suggested species. A data evaluation on the DanishFungi 2021 — data that have been submitted for automatic recognition — has shown that only 7.18% were not approved by the community or expert validation, thus revealing a far better performance than most non-expert users in the system. Almost two thirds (69.28%) of the approved species identifications were based on the highest-ranking AI suggesting species ID. In contrast, another 12.28% were based on the second-highest-ranking AI suggested species ID and another 10.54% were based on the top 3–5 suggestions. In other words, the AI system achieved the Top1 accuracy of 69.28% and the Top1 accuracy of 92.82% in combination with citizen scientists.

**Figure 7:**
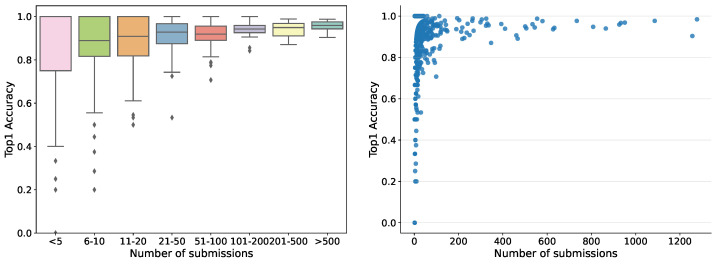
Dependence of human in the loop performance on the number of submissions. The 651 users with one submission only were filtered out.

So far, the automatic recognition system has been tested by 1769 users — each submitting between one and 1277 records — who contributed 35,018 fungi sightings over the past 2 years. For users submitting more than ten records, the accuracy in terms of correct identifications guided by the system varied from 30% to 100%, pointing to quite considerable differences in how well different users have been able to identify the correct species using the system. Hence, the tool is not fully reliable but helps non-expert users to gain better identification skills. The accuracy was variable among the fungal morphogroups defined in the fungal atlas, varying from 24% to 100% for groups with more than ten records. The accuracy was tightly correlated with the obtained morphogroup user score based on the algorithms deployed in the Atlas of Danish Fungi to support community validation. Within the first month the server ran, more than 20,000 images were submitted for recognition. The dependence of human in the loop performance on the number of submissions, for example, recognition experience, is shown in Figure 7.

### 6.3. Convolutional Neural Networks vs. Vision Transformers

In this section, we compare the performance of the well known CNN based models and ViT models in terms of Top1 and Top3 accuracy on the DF20 and the FGVCx Fungi’18 datasets and two different resolutions — 224 × 224 and 384 × 384.

Comparing well known CNN architectures on the DF20 dataset, we can see a similar behaviour to that on other datasets [11,17,18]. The best performing model on the DF20 and input resolution of 384 × 384 was SE-ResNeXt-101 with a Top1 score of 78.72%. EfficientNetV2-L achieved a slightly lower accuracy of 77.83%. On a smaller input resolution (224 × 224), the best performing model was the EfficientNetV2-L, while achieving a better performance by 1.22% than SE-ResNeXt-101.

Comparing two ViT architectures — ViT-Base/16 and ViT-Large/16 — against the well-performing CNN models — EfficientNetV2-L and SE-ResNeXt-101 — on a DF20 dataset, we see a difference from the performance evaluation on ImageNet [74,75]. In our experiments, ViTs outperform state-of-the-art CNNs by a large margin in a 384 × 384 scenario. The best performing ViT model achieved an impressive Top1 accuracy of 80.45% while outperforming the SE-ResNeXt-101 by a significant margin of 1.73% on the images with 384 × 384 input size. In a 224 × 224 scenario, netiher CNNs nor ViT showed a superior performance. A wider performance comparison is shown in Table 6.

### 6.4. Importance of the Metadata

Inspired by the common practice in mycology, we set up an experiment to show the importance of metadata for *Fungus* species identification. Using the approach described in Section 4.3, we improved performance in all measured metrics by a significant margin. We measured the performance improvement with all metadata types and their combinations. Overall, the habitat was most efficient in improving the performance. With the combination of habitat, substrate and month, we improved the ViT-Large/16 model’s performance on DF20 by 2.95% and 1.92% in Top1 and Top3, respectively, and the performance of the ViT-Base/16 model by 3.81% and 2.84% in Top1 and Top3. A detailed evaluation of the performance gain using different observation metadata and their combinations is shown in Table 7.

### 6.5. Impact on Mycology–Atlas of Danish Fungi

**Figure 8:**
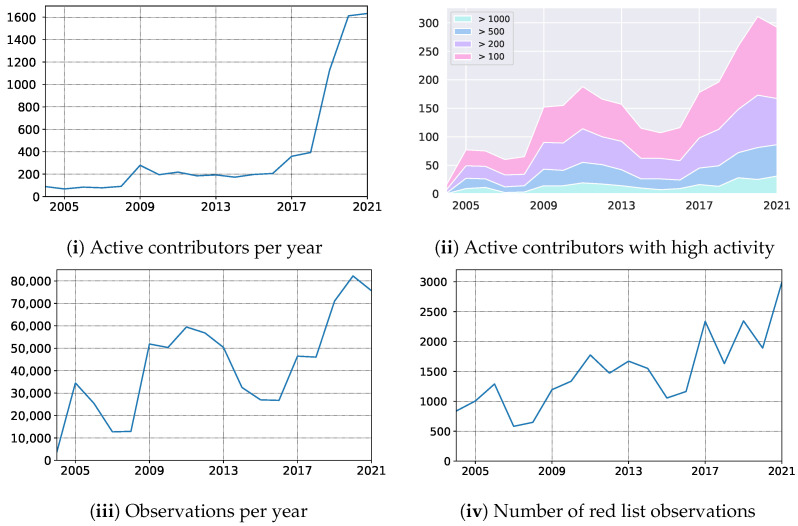
Impact on the Atlas of Danish Fungi community: the number of (**i**) active contributors, (**ii**) their distribution according to number of observations submitted, the number of (**iii**) observations, and (**iv**) observations of species from the Redlist.

In October 2019, we launched the mobile application empowering Atlas of Danish Fungi with users with an image-based recognition tool for fungi species identification. The launch received good press coverage, including an appearance in the evening news on Danish National television — TV2. The launch led to an immediate increase in the user base, increasing the number of weekly contributors from 150 to 400. Besides, the number of yearly contributors quadrupled from 2018 to 2020, resulting in a 79% increase in submitted records (Figure 8 (i) — the number of active contributors, (iii) yearly records). In parallel, the average number of records submitted per contributor dropped by 49% (from 117 to 51), indicating a substantial increase in less dedicated fungal recorders but with much broader geographical coverage. However, even the most active user groups with more than 100, 200, 500, and 1000 records a year also increased their size by 55%, 44%, 66%, and 92%, respectively (Figure 8 (ii) — number of contributors with a various number of min records).

Over a longer period, including the first very active recording period from 2009–2013, the shift from a relatively small but dedicated user group to a much larger group including more and less active contributors is even more evident.

Table 8 shows the comparison of our original FungiVision system and a newly proposed system, comprising a Vision Transformer trained on DF20 and utilizing the available metadata. With the new system, the Top-1 error in was reduced by 48.2%.

Building on the terminology suggested by Ceccaroni et al. [87], the application of AI in the Atlas of Danish Fungi has mainly contributed to influencing human behaviour, that is, attracting many new contributors, who earlier tended to find fungi too challenging to identify. Anyway, based on our yearly evaluation — see Table 9 — both the higher number of users and submissions containing more challenging species for identification did not affect the overall user performance. So far, we have not explored the educational and social benefits for new contributors in detail, but from casual oral and written responses from new contributors, the effects seem to be considerable. The large influx of new contributors has been a challenge for already associated expert users and professional experts associated with the project. The system is designed to be interactive and involving, requiring new users to be trained to submit high-quality records and contribute actively to the validation process. The development of automatic response options, for example, addressing common issues related to the poor quality of submitted photos or inadequate meta-data, could solve some of these issues in the future, potentially using AI to replace the time-consuming human evaluation of records.

### 6.6. Impact on AI–Fungi Recognition in 2021

We introduce a novel fine-grained dataset and benchmark based on the symbiotic relationship between Machine Learning and Mycology, the Danish Fungi 2020 (DF20). The dataset, constructed from observations submitted to the Atlas of Danish Fungi, is unique in its taxonomy-accurate class labels, small number of errors, highly unbalanced long-tailed class distribution, rich observation metadata, and well-defined class hierarchy. DF20 has zero overlap with ImageNet, allowing unbiased comparison of models fine-tuned from publicly available ImageNet checkpoints. The proposed evaluation protocol enables testing the ability to improve classification using metadata — for example, location, habitat, and substrate, facilitates classifier calibration testing and finally allows us to study the impact of the device settings on the classification performance.

Experimental comparison of selected CNN and ViT architectures shows that DF20 presents a challenging task. Interestingly, ViT achieves results superior to CNN baselines with 80.45% accuracy, reducing the CNN error by 9%.

A simple procedure for including metadata into the decision process improves the classification accuracy by more than 2.95 and 0.65 percentage points, reducing the error rate by 15% and 6.5% on Danish Fungi 2020 and Danish Fungi 2021, respectively.

In Table 10, we present the comparison on the FGVCx Fungi’18 test set between our novel approach where we utilize the ViT architecture and metadata, and the single model developed back in 2018. We can see a significant increase in performance by 6.59% in terms of Top1 Accuracy. Evaluated via Kaggle using 80% central crop.

To allow further research in areas of Deep Learning mentioned above, we made the source code for all methods and experiments is available at https://sites.google.com/view/danish-fungi-dataset.

## 7. Conclusions

A machine learning system for automatic fungi recognition, a winner of a computer vision Kaggle challenge, was deployed as an online recognition service to help a community of citizen scientists identify the species of an observed specimen.

The development of the machine learning system for the Kaggle competition in Section 4.1 showed the effect of calibrating outputs to new a priori probabilities, test-time data augmentation and ensembles: together, these “tricks” increased the recognition accuracy by almost 12% and helped us to score 1st in the FGVCx Fungi Classification competition hosted on Kaggle, achieving a Top3 Accuracy of 73%. The availability of the identification service helped to increase the activity and contributions of citizen scientists to the Atlas of Danish Fungi. Integration of the image recognition system into the Atlas of Danish Fungi has made community-based fungi observation identification easier: 92.82% of submissions labeled by users with the help of the FungiVision system were identified correctly.

The collected data allowed the creation of a novel fine-grained classification dataset — the Danish Fungi 2020 (DF20) — which has zero overlap with ImageNet, allowing unbiased comparison of models fine-tuned from publicly available ImageNet checkpoints. With the precise annotation and rich metadata coming with the DF20 dataset, we would like to encourage research in other areas of computer vision and machine learning, beyond fine-grained visual categorization. The datasets may serve as a benchmark for classifier calibration, loss functions, validation metrics, taxonomy and hierarchical learning, device dependency or time series based species prediction. For example, the standard loss function focusing on recognition accuracy ignores the practically important cost of predicting a species with high toxicity. The quantitative and qualitative analysis of CNNs and ViTs showed superior performance of the ViT in fine-grained classification. We present the baselines for processing the habitat, substrate and time (month) metadata. We show that — even with the simple method from Section 4.3 — utilizing the metadata increases the classification performance significantly. A new Vision Transformer architecture, trained on DF20 and exploiting available metadata, with a recognition error 46.75% lower than that of the current system.

Cross science efforts, such as the collaboration described here, can develop tools for citizen-scientists that improve their skills and the quality of the data they generate. Along with data generated by DNA sequencing, this may help by lowering the taxonomic bias in the biodiversity information data available in the future. By providing a stream of labeled data in one direction and an accuracy increase in the other, the collaboration creates a virtuous cycle, helping both communities.

## Figures and Tables

**Table 1:** The number of species and images in publicly available fungi datasets.

Dataset	Species	Training Images	Val. Images	Test Images
*iNaturalist 2021*	* 341*	* 90,048*	*3410*	×
FGVCx Fungi’18	1394	89,760	4182	9758
Danish Fungi 2020	1604	266,344	×	29,594
Danish Fungi 2021${}^{†}$	999	×	×	14,391
Atlas of Danish Fungi ${}^{‡}$	6406	×	×	255,224

^†^ Observations with species level labels submitted to Atlas of Danish Fungi in 2021; ^‡^ Observations with species level labels submitted to Atlas of Danish Fungi since 2018

**Table 5:** Results of the top five teams in the FGVCx Fungi Classification Challenge.

		Private Test Set	Public Test Set
Rank	Team Name	Top3 [%]	Top3 [%]
1	our method described in Section 4.1	**78.80**	**79.23**
2	digitalspecialists	76.81	76.53
3	Val An	74.91	74.79
4	DL Analytics	71.66	73.15
5	Invincibles	71.25	71.51

**Table 6:** Top1 and Top3 accuracy of selected CNN and ViT architectures on the DF20 dataset for two resolutions. For 384 × 384, ViT is superior to CNNs achieving 80.45% Top1, reducing the error of the best CNN by 9%. On 224 × 224, the ViT and EfficientNetV2-L differ insignificantly.

	Top1 [%]	Top3 [%]	Top1 [%]	Top3 [%]
Inception-ResNet-V2	69.53	84.30	75.43	88.58
EfficientNet-B3	72.51	86.77	77.59	90.07
EfficientNetV2-L	**75.48**	**88.38**	77.83	89.59
SE-ResNeXt-101	74.26	87.78	78.72	90.54
ViT-Base/16	73.51	87.55	79.48	90.95
ViT-Large/16	75.29	88.34	**80.45**	**91.68**
**Image Resolution** **—>**	**224 × 224**		**384 × 384**	

**Table 7:** Performance gains based on three observation metadata and their combination. Tested on the Danish Fungi 2020 dataset with different ViT architectures. H — Habitat, S — Substrate, M — Month.

			ViT-Large/16—384×384		ViT-Base/16—224×224	
H	M	S	Top1 [%]	Top3 [%]	Top1 [%]	Top3 [%]
×	×	×	80.45	91.68	73.51	87.55
✔	×	×	+1.50	+1.00	+1.94	+1.50
×	✔	×	+0.95	+0.62	+1.23	+0.95
×	×	✔	+1.13	+0.69	+1.39	+1.17
×	✔	✔	+1.93	+1.27	+2.47	+1.98
✔	×	✔	+2.48	+1.66	+3.23	+2.47
✔	✔	×	+2.31	+1.48	+3.11	+2.30
✔	✔	✔	+**2.95**	+**1.92**	+**3.81**	+**2.84**

**Table 8:** Comparison of DNN models and the AI + Human approach on Danish Fungi 2021.

Approach	Training Data	Metadata	Top1 [%]	Top5 [%]
Inception-ResNet-v2	Fungi’18	×	69.28	92.10
ViT/Base-16	DF20	×	82.11	93.79
ViT/Large-16	DF20	×	82.99	93.77
ViT/Base-16	DF20	✔	82.85	94.03
ViT/Large-16	DF20	✔	83.64	94.05
			Acc	mean Acc
*Human in the Loop* with Inception-ResNet-v2 (Fungi’18)			**92.82**	**87.1**

**Table 9:** Comparison of Fungi species recognition ability for different Atlas of Danish Fungi users.

			User Recognition Ability [%]			
Year	# of Observations	# of Users	Citizen Scientists		Experts	
2018	49,826	377	94.65	**92.62**	97.20	95.84
2019	67,009	913	94.31	88.15	96.49	95.92
2020	76,812	1,293	94.42	88.03	97.55	93.97
2021	72,965	1,326	**95.33**	88.46	**97.63**	**96.99**
Performance metric —>			ACC	mean ACC	ACC	mean ACC

**Table 10:** Top3 accuracy for two DNN architectures on the FGVCx Fungi’18 test set.

Architecture	Training Data	Input Size	Private	Public
Inception-ResNet-v2	FGVCx Fungi’18	299 × 299	70.69	71.37
ViT/Large-16	DF20	384 × 384	**77.28**	**76.60**

## Data Availability

The source code is available through the GitHub repositories: https://github.com/sulc/fungi-recognition and https://github.com/picekl/DanishFungiDataset. The data and neural network checkpoints are available throuth the Dataset Web https://sites.google.com/view/danish-fungi-dataset.

## Abbreviations

The following abbreviations are used in this manuscript:

DNN	Deep Neural Network
CNN	Convolutional Neural Network
DCNN	Deep Convolutional Neural Network
NN	Neural Network
ViT	Vision Transformer
REST	Representational State Transfer
API	Application Programming Interface
AI	Artificial Intelligence
GBIF	Global Biodiversity Information Facility
FGVC	Fine Grained Visual Categorization

## References

[1] Dickinson J.L., Shirk J., Bonter D., Bonney R., Crain R.L., Martin J., Phillips T., Purcell K. (2012). The current state of citizen science as a tool for ecological research and public engagement. Front. Ecol. Environ..

[2] Jetz W., McGeoch M.A., Guralnick R., Ferrier S., Beck J., Costello M.J., Fernandez M., Geller G.N., Keil P., Merow C. (2019). Essential biodiversity variables for mapping and monitoring species populations. Nat. Ecol. Evol..

[3] Andrew C., Heegaard E., Kirk P.M., Bässler C., Heilmann-Clausen J., Krisai-Greilhuber I., Kuyper T.W., Senn-Irlet B., Büntgen U., Diez J. (2017). Big data integration: Pan-European fungal species observations’ assembly for addressing contemporary questions in ecology and global change biology. Fungal Biol. Rev..

[4] van Strien A.J., Boomsluiter M., Noordeloos M.E., Verweij R.J.T., Kuyper T.W. (2018). Woodland ectomycorrhizal fungi benefit from large-scale reduction in nitrogen deposition in the Netherlands. J. Appl. Ecol..

[5] Heilmann-Clausen J., Maruyama P.K., Bruun H.H., Dimitrov D., Læssøe T., Frøslev T.G., Dalsgaard B. (2016). Citizen science data reveal ecological, historical and evolutionary factors shaping interactions between woody hosts and wood-inhabiting fungi. New Phytol..

[6] Bayraktarov E., Ehmke G., O’connor J., Burns E.L., Nguyen H.A., McRae L., Possingham H.P., Lindenmayer D.B. (2019). Do big unstructured biodiversity data mean more knowledge?. Front. Ecol. Evol..

[7] Chandler M., See L., Copas K., Bonde A.M.Z., López B.C., Danielsen F., Legind J.K., Masinde S., Miller-Rushing A.J., Newman G. (2017). Contribution of citizen science towards international biodiversity monitoring. Biol. Conserv..

[8] Dickinson J., Zuckerberg B., Bonter D.N. (2010). Citizen Science as an Ecological Research Tool: Challenges and Benefits. Annu. Rev. Ecol. Evol. Syst..

[9] Geldmann J., Heilmann-Clausen J., Holm T.E., Levinsky I., Markussen B., Olsen K., Rahbek C., Tøttrup A.P. (2016). What determines spatial bias in citizen science? Exploring four recording schemes with different proficiency requirements. Divers. Distrib..

[10] iNaturalist. http://www.inaturalist.org.

[11] Deng J., Dong W., Socher R., Li L.J., Li K., Fei-Fei L. ImageNet: A large-scale hierarchical image database. Proceedings of the 2009 IEEE Conference on Computer Vision and Pattern Recognition.

[12] Russakovsky O., Deng J., Su H., Krause J., Satheesh S., Ma S., Huang Z., Karpathy A., Khosla A., Bernstein M. (2015). ImageNet Large Scale Visual Recognition Challenge. Int. J. Comput. Vis..

[13] Everingham M., Van Gool L., Williams C.K.I., Winn J., Zisserman A. (2010). The Pascal Visual Object Classes (VOC) Challenge. Int. J. Comput. Vis..

[14] Goëau H., Bonnet P., Joly A. Plant identification in an open-world (LifeCLEF 2016). Proceedings of the CLEF: Conference and Labs of the Evaluation Forum.

[15] Goeau H., Bonnet P., Joly A. Plant identification based on noisy web data: The amazing performance of deep learning (LifeCLEF 2017). Proceedings of the CLEF: Conference and Labs of the Evaluation Forum.

[16] Joly A., Goëau H., Glotin H., Spampinato C., Bonnet P., Vellinga W.P., Planqué R., Rauber A., Palazzo S., Fisher B. (2015). LifeCLEF 2015: Multimedia life species identification challenges. Proceedings of the International Conference of the Cross-Language Evaluation Forum for European Languages.

[17] Van Horn G., Mac Aodha O., Song Y., Cui Y., Sun C., Shepard A., Adam H., Perona P., Belongie S. The inaturalist species classification and detection dataset. Proceedings of the IEEE Conference on Computer Vision and Pattern Recognition, Salt Lake City.

[18] Wah C., Branson S., Welinder P., Perona P., Belongie S. (2011). The Caltech-UCSD Birds-200-2011 Dataset.

[19] Nilsback M.E., Zisserman A. Automated Flower Classification over a Large Number of Classes. Proceedings of the 2008 Sixth Indian Conference on Computer Vision, Graphics & Image Processing.

[20] Gebru T., Hoffman J., Fei-Fei L. Fine-grained recognition in the wild: A multi-task domain adaptation approach. Proceedings of the IEEE International Conference on Computer Vision.

[21] He K., Fan H., Wu Y., Xie S., Girshick R. Momentum Contrast for Unsupervised Visual Representation Learning. Proceedings of the IEEE/CVF Conference on Computer Vision and Pattern Recognition (CVPR).

[22] Oh Song H., Xiang Y., Jegelka S., Savarese S. Deep metric learning via lifted structured feature embedding. Proceedings of the IEEE Conference on Computer Vision and Pattern Recognition.

[23] Sohn K. Improved deep metric learning with multi-class n-pair loss objective. Proceedings of the 30th International Conference on Neural Information Processing Systems.

[24] Zhai A., Wu H.Y. Classification is a strong baseline for deep metric learning. Proceedings of the British Machine Vision Conference.

[25] Li D., Hung W.C., Huang J.B., Wang S., Ahuja N., Yang M.H. (2016). Unsupervised visual representation learning by graph-based consistent constraints. European Conference on Computer Vision.

[26] Lorenz D., Bereska L., Milbich T., Ommer B. Unsupervised part-based disentangling of object shape and appearance. Proceedings of the IEEE/CVF Conference on Computer Vision and Pattern Recognition.

[27] Wertheimer D., Hariharan B. Few-shot learning with localization in realistic settings. Proceedings of the IEEE/CVF Conference on Computer Vision and Pattern Recognition.

[28] Sulc M., Matas J. Improving CNN Classifiers by Estimating Test-Time Priors. Proceedings of the IEEE/CVF International Conference on Computer Vision (ICCV) Workshops.

[29] Sulc M., Picek L., Matas J., Jeppesen T., Heilmann-Clausen J. Fungi Recognition: A Practical Use Case. Proceedings of the IEEE/CVF Winter Conference on Applications of Computer Vision (WACV).

[30] Picek L., Šulc M., Matas J., Heilmann-Clausen J., Jeppesen T.S., Læssøe T., Frøslev T. Danish Fungi 2020–Not Just Another Image Recognition Dataset. Proceedings of the IEEE/CVF Winter Conference on Applications of Computer Vision (WACV).

[31] Krizhevsky A., Sutskever I., Hinton G.E. Imagenet classification with deep convolutional neural networks. Proceedings of the Advances in Neural Information Processing Systems.

[32] Zhang N., Donahue J., Girshick R., Darrell T. (2014). Part-based R-CNNs for fine-grained category detection. Computer Vision—ECCV 2014, Proceedings of the European Conference on Computer Vision, Zurich, Switzerland, 6–12 September 2014.

[33] Huang S., Xu Z., Tao D., Zhang Y. Part-stacked cnn for fine-grained visual categorization. Proceedings of the IEEE Conference on Computer Vision and Pattern Recognition.

[34] Feng Z., Fu K., Zhao Q. Learning to focus and discriminate for fine-grained classification. Proceedings of the 2019 IEEE International Conference on Image Processing (ICIP).

[35] Xiao T., Xu Y., Yang K., Zhang J., Peng Y., Zhang Z. The application of two-level attention models in deep convolutional neural network for fine-grained image classification. Proceedings of the IEEE Conference on Computer Vision and Pattern Recognition.

[36] Liu X., Xia T., Wang J., Yang Y., Zhou F., Lin Y. (2017). Fully Convolutional Attention Networks for Fine-Grained Recognition. arXiv.

[37] Lin T.Y., RoyChowdhury A., Maji S. Bilinear cnn models for fine-grained visual recognition. Proceedings of the IEEE International Conference on Computer Vision.

[38] Zhang L., Huang S., Liu W., Tao D. Learning a Mixture of Granularity-Specific Experts for Fine-Grained Categorization. Proceedings of the IEEE/CVF International Conference on Computer Vision (ICCV).

[39] Cui Y., Song Y., Sun C., Howard A., Belongie S. Large scale fine-grained categorization and domain-specific transfer learning. Proceedings of the IEEE Conference on Computer Vision and Pattern Recognition, Salt Lake City.

[40] Ngiam J., Peng D., Vasudevan V., Kornblith S., Le Q.V., Pang R. (2018). Domain Adaptive Transfer Learning with Specialist Models. arXiv.

[41] Goëau H., Bonnet P., Joly A. Overview of ExpertLifeCLEF 2018: How far automated identification systems are from the best experts? In Proceedings of the CLEF-Conference and Labs of the Evaluation Forum, Avignon, France, 10–14 September 2018.

[42] Goëau H., Bonnet P., Joly A. Overview of LifeCLEF Plant Identification task 2019: Diving into data deficient tropical countries. Proceedings of the CLEF 2019-Conference and Labs of the Evaluation Forum.

[43] Šulc M., Matas J. (2017). Fine-grained recognition of plants from images. Plant Methods.

[44] Carranza-Rojas J., Goeau H., Bonnet P., Mata-Montero E., Joly A. (2017). Going deeper in the automated identification of Herbarium specimens. BMC Evol. Biol..

[45] Khosla A., Jayadevaprakash N., Yao B., Fei-Fei L. Novel Dataset for Fine-Grained Image Categorization. Proceedings of the First Workshop on Fine-Grained Visual Categorization, IEEE Conference on Computer Vision and Pattern Recognition.

[46] Picek L., Durso A., De Castañeda R.R., Bolon I. Overview of SnakeCLEF 2021: Automatic snake species identification with country-level focus. Proceedings of the CEUR Workshop Proceedings.

[47] Welinder P., Branson S., Mita T., Wah C., Schroff F., Belongie S., Perona P. (2010). Caltech-UCSD Birds 200.

[48] Wei X.S., Xie C.W., Wu J., Shen C. (2018). Mask-CNN: Localizing parts and selecting descriptors for fine-grained bird species categorization. Pattern Recognit..

[49] Joly A., Goëau H., Kahl S., Deneu B., Servajean M., Cole E., Picek L., De Castaneda R.R., Bolon I., Durso A. (2020). Overview of LifeCLEF 2020: A system-oriented evaluation of automated species identification and species distribution Prediction. International Conference of the Cross-Language Evaluation Forum for European Languages.

[50] Joly A., Goëau H., Kahl S., Picek L., Lorieul T., Cole E., Deneu B., Servajean M., Durso A., Bolon I. (2021). Overview of lifeclef 2021: An evaluation of machine-learning based species identification and species distribution prediction. International Conference of the Cross-Language Evaluation Forum for European Languages.

[51] (1999). Global Biodiversity Information Facility. https://www.gbif.org.

[52] Robertson T., Belongie S., Adam H., Kaeser-Chen C., Zhang C., Tan K.C., Liu Y., Brulé D., Deltheil C., Loarie S. (2019). Training Machines to Identify Species using GBIF-mediated Datasets. Biodivers. Inf. Sci. Stand..

[53] (2019). iNaturalist.org. iNaturalist Research-Grade Observations. https://www.gbif.org/dataset/50c9509d-22c7-4a22-a47d-8c48425ef4a7.

[54] Parham J., Stewart C., Crall J., Rubenstein D., Holmberg J., Berger-Wolf T. An Animal Detection Pipeline for Identification. Proceedings of the 2018 IEEE Winter Conference on Applications of Computer Vision (WACV).

[55] (2009). Danish Mycological Society. https://svampe.databasen.org.

[56] Frøslev T.G., Heilmann-Clausen J., Lange C., Læssøe T., Petersen J.H., Søchting U., Jeppesen T.S., Vesterholt J. (2021). Danish Mycological Society, Fungal Records Database. https://www.gbif.org/dataset/84d26682-f762-11e1-a439-00145eb45e9a.

[57] Heilmann-Clausen J., Bruun H.H., Ejrnæs R., Frøslev T.G., Læssøe T., Petersen J.H. (2019). How citizen science boosted primary knowledge on fungal biodiversity in Denmark. Biol. Conserv..

[58] Tahir M.W., Zaidi N.A., Rao A.A., Blank R., Vellekoop M.J., Lang W. (2018). A Fungus Spores Dataset and a Convolutional Neural Network Based Approach for Fungus Detection. IEEE Trans. Nanobiosci..

[59] Zhang J., Lu S., Wang X., Du X., Ni G., Liu J., Liu L., Liu Y. (2017). Automatic identification of fungi in microscopic leucorrhea images. J. Opt. Soc. Am. A.

[60] Zieliński B., Sroka-Oleksiak A., Rymarczyk D., Piekarczyk A., Brzychczy-Włoch M. (2020). Deep learning approach to describe and classify fungi microscopic images. PLoS ONE.

[61] Van De Vooren J., Polder G., Van der Heijden G. (1992). Identification of mushroom cultivars using image analysis. Trans. ASAE.

[62] Fricker M., Boddy L., Bebber D. (2007). Network organisation of mycelial fungi. Biology of the Fungal Cell.

[63] Heilmann-Clausen J., Frøslev T.G., Læssøe T., Petersen J.H. (2019). Danmarks Svampeatlas 2009–2013.

[64] (2021). Occdownload Gbif.Org. Occurrence Download. https://www.gbif.org/occurrence/download/0030066-210914110416597.

[65] Szegedy C., Ioffe S., Vanhoucke V., Alemi A.A. Inception-v4, inception-resnet and the impact of residual connections on learning. Proceedings of the Thirty-First AAAI Conference on Artificial Intelligence.

[66] Guadarrama S., Silberman N. (2016). TensorFlow-Slim: A lightweight library for defining, training and evaluating complex models in TensorFlow. https://github.com/google-research/tf-slim.

[67] Polyak B.T., Juditsky A.B. (1992). Acceleration of stochastic approximation by averaging. SIAM J. Control Optim..

[68] Du Plessis M.C., Sugiyama M. (2014). Semi-supervised learning of class balance under class-prior change by distribution matching. Neural Netw..

[69] Saerens M., Latinne P., Decaestecker C. (2002). Adjusting the outputs of a classifier to new a priori probabilities: A simple procedure. Neural Comput..

[70] Danish Mycological Society, Google Research.Danish Mycological Society Fungi Embedding Model. TensorFlow Hub. https://tfhub.dev/google/svampeatlas/vision/embedder/fungi_V1/1.

[71] Hu J., Shen L., Sun G. Squeeze-and-Excitation Networks. Proceedings of the IEEE Conference on Computer Vision and Pattern Recognition, Salt Lake City.

[72] Xie S., Girshick R., Dollár P., Tu Z., He K. Aggregated residual transformations for deep neural networks. Proceedings of the IEEE Conference on Computer Vision and Pattern Recognition.

[73] Tan M., Le Q. Efficientnet: Rethinking model scaling for convolutional neural networks. Proceedings of the International Conference on Machine Learning.

[74] Tan M., Le Q.V. (2021). Efficientnetv2: Smaller models and faster training. arXiv.

[75] Dosovitskiy A., Beyer L., Kolesnikov A., Weissenborn D., Zhai X., Unterthiner T., Dehghani M., Minderer M., Heigold G., Gelly S. An Image is Worth 16 × 16 Words: Transformers for Image Recognition at Scale. Proceedings of the International Conference on Learning Representations.

[76] Vaswani A., Shazeer N., Parmar N., Uszkoreit J., Jones L., Gomez A.N., Kaiser Ł., Polosukhin I. Attention is all you need. Proceedings of the Advances in Neural Information Processing Systems.

[77] Paszke A., Gross S., Massa F., Lerer A., Bradbury J., Chanan G., Killeen T., Lin Z., Gimelshein N., Antiga L., Wallach H., Larochelle H., Beygelzimer A., d’Alché-Buc F., Fox E., Garnett R. (2019). PyTorch: An Imperative Style, High-Performance Deep Learning Library. Advances in Neural Information Processing Systems 32.

[78] Buslaev A., Iglovikov V.I., Khvedchenya E., Parinov A., Druzhinin M., Kalinin A.A. (2020). Albumentations: Fast and Flexible Image Augmentations. Information.

[79] Berg T., Liu J., Lee S.W., Alexander M.L., Jacobs D.W., Belhumeur P.N. Birdsnap: Large-Scale Fine-Grained Visual Categorization of Birds. Proceedings of the 2014 IEEE Conference on Computer Vision and Pattern Recognition.

[80] Guo C., Pleiss G., Sun Y., Weinberger K.Q. On calibration of modern neural networks. Proceedings of the International Conference on Machine Learning.

[81] Vaicenavicius J., Widmann D., Andersson C., Lindsten F., Roll J., Schön T. Evaluating model calibration in classification. Proceedings of the 22nd International Conference on Artificial Intelligence and Statistics.

[82] Olston C., Fiedel N., Gorovoy K., Harmsen J., Lao L., Li F., Rajashekhar V., Ramesh S., Soyke J. (2017). Tensorflow-serving: Flexible, high-performance ML serving. arXiv.

[83] ImageVision. https://svampe.databasen.org/en/imagevision.

[84] Danmarks SvampeAtlas—iOS App. https://apps.apple.com/us/app/danmarks-svampeatlas/id1467728588?l=da.

[85] Danmarks SvampeAtlas—Android App. https://play.google.com/store/apps/details?id=com.noque.svampeatlas&hl=en&gl=US.

[86] Googles ML Kit. https://developers.google.com/ml-kit.

[87] Ceccaroni L., Bibby J., Roger E., Flemons P., Michael K., Fagan L., Oliver J.L. (2019). Opportunities and risks for citizen science in the age of artificial intelligence. Citiz. Sci. Theory Pract..

